# Evaluating metabolic changes in H9c2 cardiomyoblasts exposed to LPS: Towards understanding sepsis mechanisms

**DOI:** 10.1371/journal.pone.0334234

**Published:** 2025-10-09

**Authors:** Amandine Szczesnowski, Karine Pichavant-Rafini, Marie-Dominique Jezequel, Michaël Théron, Karelle Léon

**Affiliations:** ORPHY, Univ Brest, Brest, France; Emory University School of Medicine, UNITED STATES OF AMERICA

## Abstract

Sepsis is a major public health concern associated with high mortality rates, particularly due to sepsis-induced myocardial dysfunction (SIMD), which affects about 50% of septic patients. This study investigates how mitochondrial dysfunction contributes to SIMD by examining metabolic changes in H9c2 cardiomyoblasts exposed to varying concentrations of lipopolysaccharide (LPS), a bacterial endotoxin, to enhance our understanding of the relationship between infection severity and metabolic responses. H9c2 cells were treated with LPS at concentrations of 0.5, 1, 2.5, and 5 µg/mL for 24 or 48 hours. Cell viability was measured using the MTT assay, and gene expression related to inflammation and mitochondrial function was analyzed through Real-Time PCR. Mitochondrial respiration and energy metabolism were assessed using the Seahorse MitoStress kit. Results showed that while 2.5 and 5 µg/mL of LPS for 24 hours did not significantly impact cell viability, exposure to 5 µg/mL for 48 hours led to a 77.7% decrease in survival. Gene analysis indicated significant overexpression of *IL-6* and *SOD2*, with consistent underexpression of *mt-ND1*. Mitochondrial respiration increased at lower LPS concentrations but decreased at 5 µg/mL. Glycolytic metabolism also increased at lower LPS levels but decreased at higher concentrations. Inhibition of metabolic pathways affected mitochondrial function, especially at higher LPS concentrations. Our findings suggest that LPS induces metabolic disturbances in H9c2 cells, with adaptive responses at lower concentrations. However, excessive exposure results in mitochondrial and cellular damage, decreasing overall metabolism.

## 1 Introduction

Sepsis, defined since 2016 as organ dysfunction resulting from an inappropriate response to infection [1], is a critical public health issue. In 2017, there were approximately 49 million cases, leading to 11 million deaths, which accounted for 20% of global mortality [2]. Among its complications, sepsis-induced myocardial dysfunction (SIMD) is particularly concerning, as it affects about 50% of septic patients and is associated with increased mortality [3]. While there is no universal definition for SIMD, it encompasses various cardiac dysfunctions, including reduced left ventricular ejection fraction and impaired diastolic function [4]. These impairments often coincide with shifts in energy substrate utilization, further diminishing the heart’s ATP-generating capacity.

The pathophysiology of SIMD involves complex interactions among extracellular and intracellular mechanisms, including calcium dysregulation, autonomic dysfunction, oxidative stress, altered metabolism, and mitochondrial dysfunctions [5–10]. Mitochondrial dysfunctions are central in SIMD, characterized by structural changes, altered electron transport chain activity, and increased reactive oxygen species (ROS) production [6]. Given mitochondria’s essential role in energy metabolism, understanding cardiac metabolism in SIMD is vital. The heart, with its high energy demands, primarily generates ATP through oxidative phosphorylation (95%) and to a lesser extent from glycolysis (5%) [11]. Under normal conditions, 70% to 90% of cardiac ATP is produced via fatty acid oxidation, while glucose oxidation contributes 10% to 30%, facilitated by glucose transporters (GLUT) [12]. Disruptions in these processes can significantly impair myocardial contractility [13].

Sepsis triggers an uncontrolled systemic inflammatory response, characterized by phases of hyperinflammation [14] and microvascular dysfunction, leading to tissue hypoxia [15], which can disrupt mitochondrial metabolism. This complexity complicates *in vivo* studies aimed at pinpointing the origins of mitochondrial dysfunction in SIMD. To explore the metabolic changes associated with SIMD due to pathogen exposure, we utilized the H9c2 cardiomyoblast model, exposing the cells to various concentrations of LPS to simulate clinical conditions encountered in sepsis. This approach enhances the representativeness of our findings and deepens our understanding of the underlying mechanisms, considering the variability in disease severity.

Our objectives are to (1) investigate mitochondrial gene expression and the balance between oxidative and glycolytic metabolism in H9c2 cells during inflammation, and (2) determine the primary energy substrate utilized by these cells in an inflammatory environment. This research aims to enhance the intricate relationship between mitochondrial dysfunction and energy metabolism in the context of sepsis.

## 2 Materials and methods

### 2.1 Cell culture

The H9c2 cell line (CRL-1446, ATCC), derived from the ventricular tissue of embryonic rat heart, was cultured in modified Eagle’s medium Dulbecco – Ham F12 (DMEM-F12; L0093-500, BioWest) containing 10% (v:v) fetal bovine serum (FBS; S1810-500 BioWest) and 1% (v:v) penicillin and streptomycin (P/S; DE17-602E, LONZA). The cells were maintained at 37°C in a humidified atmosphere of 95% air and 5% CO_2_. The medium was changed every 2–3 days, and the cells were passaged when a confluence of 80–90% was reached. Manipulations were performed on cells with fewer than 20 passages.

### 2.2 Exposure to LPS

After seeding, the cells were allowed to adhere for 24 hours. The medium was replaced with DMEM-F12 supplemented with 1% (v:v) FBS and 1% P/S for a duration of 24 hours. Based on literature data [16–19] and considering excessively high concentrations are likely to lead to significant cell mortality, hindering the study of metabolism, the cells were treated for 24 or 48 hours without LPS or with 0.5, 1, 2.5, and 5 µg/mL of LPS diluted in DMEM-F12 1% P/S without FBS, and incubated at 37°C, 95% air, 5% CO_2_ in a humidified environment.

### 2.3 Cell viability test

Cell viability was assessed using the MTT assay (M5655, Sigma Aldrich). Cells were seeded at 6,000 cells/well in a 96-well plate and treated without LPS or with LPS (2.5, 5 µg/mL) for 24 or 48 hours. The medium was then removed and replaced with 100 µL per well of a solution of MTT reconstituted at 0.05% in DMEM-F12 1% P/S, 0% FBS. The cells were incubated for 2 hours at 37°C and 5% CO_2_ in the dark. The supernatant was removed, and dimethyl sulfoxide (DMSO, D4540, Sigma Aldrich) was added for 15 minutes in each well to lyse the cell membranes and dissolve the formed formazan crystals. Absorbance was measured at 570 nm with background noise at 690 nm using a plate reader (Xenius XM Spectrofluorometer, SAFAS). Optical density (OD) measurements were reported relative to the control group, which was set at 100%.

### 2.4 Gene expression

#### 2.4.1 RNA isolation for RT-PCR.

Total RNA was isolated using the NucleoZOL RNA Kit NucleoSpin™ (740406.50, Macherey-Nagel). Briefly, a cell pellet of 2 × 10^5^ H9c2 cells was recovered in 500 µL of NucleoZOL using a 20G needle. Then, 200 μL of DNase/RNase-free water was added to the lysate. The mixture was incubated for 15 minutes at room temperature and then centrifuged (15 min, 12,000 G, 4°C). 600 µL of supernatant was collected, and an equivalent volume of mix buffer was added. This mix was transferred to a NucleoSpin® column and centrifuged (1 min, 8,000 G, room temperature). After this step, the RNA was bound to the silica membrane. Subsequently, two washes were performed with the wash buffer (RA3) provided in the kit. RNA was eluted in 30 µL of DNase/RNase-free water and recovered after centrifugation (1 min, 11,000 G, room temperature). It was then stored at −80°C. The concentration of messenger RNA was determined using the SimpliNano™ spectrophotometer (29-0617-12, GE Healthcare Life Sciences). The purity of the sample was evaluated using the optical density (OD) ratios 260 nm/OD 280 nm, with threshold values ranging from 1.8 to 2.1. The integrity of the RNA was verified by electrophoresis on a 1.5% (m: v) agarose gel (A9539, Sigma Aldrich) containing ethidium bromide.

#### 2.4.2 Quantification of gene expression by Real-Time Reverse Transcriptase-PCR (RT-PCR).

Reverse transcription was performed following the manufacturer’s recommendations for the qScript™ cDNA synthesis kit (733−1174, QuantaBio), using 1 ng of RNA. The reverse transcription was carried out according to the following program: 1 cycle at 22°C for 5 min, 1 cycle at 42°C for 30 min, 1 cycle at 85°C for 5 min, end at 4°C. The obtained cDNA was diluted 1:10 in DNase/RNase-free water and stored at −20°C until analysis.

Analyses were performed using the 7500 Fast Real-Time PCR system (Applied Biosystems, Thermo Fisher Scientific). Amplification of target genes was conducted using specific primers (Table 1), and quantification was performed by incorporating SYBR® Green (PB20.11−05, Eurobio). A denaturation step at 95°C was conducted for 2 minutes, followed by 50 amplification cycles (denaturation: 5 seconds at 95 °C; hybridization/extension: 30 seconds at 60°C). Each gene (*IL-6, SOD2, mt-ND1, Sirt3, Fis1, OPA1, Mfn1, Mfn2, Drp1, mt-Cox2, mt-Cox4i1, mt-ATP6,* β-actin) was amplified in duplicate. *IL-6* was used for inflammatory stress and activation of the NFkB pathway; *SOD2, Sirt3, Fis1,* and *Drp1* for oxidative stress response and mitochondrial dynamics, with *Fis1* and *Drp1* indicating cellular stress; *OPA1, Mfn1, Mfn2* for mitochondrial fusion genes and *mt-ND1, mt-Cox2, mt-Cox4i1, mt-ATP6* for subunits of the electron transport chain. Data were normalized using *β-actin* as a reference gene (Table 1). This choice was supported by the absence of significant differences in *β-actin* RNA levels between the different experimental groups (*p-value > 0.05*). Data analysis was performed by comparing the relative expression of mRNA using the 2-^ΔΔCt^ method [20].

**Table 1:** Primer sequences used for Real-Time RT-PCR analysis.

Target gene	Primer sequence (5’ to 3’)	Reverse sequence (5’ to 3’)	Accession number	Data base
*IL-6*	CTCAGGGAGATCTTGGAAATG	CCAGTTTGGAAGCATCCATCA	NM_012589.2	134
*SOD2*	TGGCTTGGCTTCAATAAGGAG	AAGATAGTAAGCGTGCTCCCA	NM_017051.2	129
*mt-ND1*	CGCCTGACCAATAGCCATAAT	TTCGACGTTAAAGCCTGAGAC	ENSRNOT00000047550.4	112
*Sirt 3*	CTCATGGGTCCTTTGTATCAG	TCAGGTTTCACAACGCCAGTA	NM_001106313.2	130
*Fis1*	ACGCCTGCCGTTACTTCTTC	GCAACCCTGCAATCCTTCAC	XM_006249122.3	108
*OPA 1*	GGCACTTCAAGGTCGTCTCA	CACTGCTCTTGGGTCCGATT	NM_133585.3	108
*Mfn 1*	ATCTGGTGGAGATACAGGGCT	TCCCACAGCATTGCGTTGAT	NM_138976.1	136
*Mfn 2*	GCTCAGTCGGTTGGAAGTCA	GAAAGGAGTGCCTGCCTGAT	NM_130894.4	108
*Drp 1*	AGGTTGCCCGTGACAAATGA	CACAGGCATCAGCAAAGTCG	NM_053655.3	94
*mt-Cox2*	AATCTCATCCGAAGACGTCCT	GTCACTGTAGCTTGGTTTAGG	ENSRNOT00000043693.3	96
*mt-Cox4i1*	CCTGAAGGAGAAGGAGAAGG	ACTCATTGGTGCCCTTGTTCA	NM_017202.1	116
*mt-ATP6*	CCTATGAGCAGGAGCCGTAA	TGGGAATTAGGGAGATGGGG	ENSRNOT00000046108.3	98
*β-actin*	CTACAATGAGCTGCGTGTGG	GGATGGCTACGTACATGGCT	NM_031144.3	140

### 2.5 Mitochondrial respiration and energetic metabolism

Mitochondrial respiration was evaluated using the Seahorse MitoStress kit (Agilent, Cat. No. 103010−100) following the manufacturer’s instructions. H9c2 cells were seeded in Seahorse assay microplates with 180 μL of growth medium and incubated overnight. The day before the assay, an XF cartridge was hydrated with XF calibrant (Agilent, Cat. No. 103059−000) and incubated overnight at 37 °C in a non-CO_2_ incubator. Prior to the assay, the culture media was changed to 180 μL/well of XF DMEM supplemented with 10 mM glucose, 2 mM glutamine, and 1 mM pyruvate (Agilent, Cat. Nos. 103575−100, 103577−100, 103579−100, 103578−100) and incubated for 1 hour at 37 °C (without CO_2_). The oxygen consumption rate (OCR) and extracellular acidification rate (ECAR) were measured using the XF HS Seahorse Bioanalyzer (Agilent Technologies, Santa Clara, CA, USA). Obtained values were normalized to 1,000 cells by staining the nuclei with 4 µg/mL of Hoechst, incubated for 15 minutes in the dark at 37 °C. Counting was performed using the cell imaging software of the Cytation (Cytation 1, Agilent Technologies). The normalized OCR and ECAR data were reported relative to the control group, which was set at 100%. The final concentrations of injected compounds were 1 μM oligomycin, 5 μM carbonyl cyanide-4(trifluoromethoxy)phenylhydrazone (FCCP), and 1 μM rotenone + antimycin A. From the values obtained, mitochondrial respiration and energetic metabolism were measured and calculated on H9c2 cells and more particularly Basal OCR, OCR related to ATP production, Maximal OCR, Coupling efficiency, Proton leak, Reserve capacity, Basal extracellular acidification, Glycolytic capacity, Glycolytic reserve and mitochondrial ATP/Glycolytic ATP ratio. Data were reported relative to the control group, which was set at 100%.

The utilization of metabolic substrates by the mitochondria was determined using the Seahorse XF Substrate Oxidation Stress Test Kits (Agilent, Cat. Nos. 103672−100, 103673−100, 103674−100). Briefly, cells were seeded and exposed to LPS as described above (paragraph 2.2). On the day of the assay, the culture media was changed to XF DMEM supplemented with 10 mM glucose, 2 mM glutamine, and 1 mM pyruvate (Agilent, Cat. Nos. 103575−100, 103577−100, 103579−100, 103578−100) without FBS. Various metabolic pathway inhibitors were used (final concentrations): Etomoxir (4 µM; irreversibly blocks CPT1a, thereby inhibiting the fatty acid pathway), UK5099 (2 µM; inhibits the mitochondrial pyruvate transporter and prevents glycolysis) and BPTES (3 µM; inhibits glutaminase 1 and blocks the amino acid pathway) prior to exposure to the mitochondrial inhibitors oligomycin, FCCP, rotenone and antimycin A.

The concentrations of these inhibitors were determined by the manufacturer based on their tests and the literature [21–23].

### 2.6 Statistical analysis

Statistical analyses were performed using Graphpad Prism 9.0.2 software. Results are presented as mean ± SEM or median and interquartile range.

After checking for normality using the Shapiro-Wilk test and verifying homogeneity of variances, a non-parametric Kruskal-Wallis test followed by Dunn’s post hoc test was applied for comparisons among more than two conditions. For comparisons between two conditions, the Mann-Whitney test was conducted. Differences were considered significant for a *p-value* less than 0.05.

Each experimental condition included 3–8 biological replicates, each performed with 2–5 technical replicates.

## 3 Results

### 3.1 Cell viability

**Fig 1:**
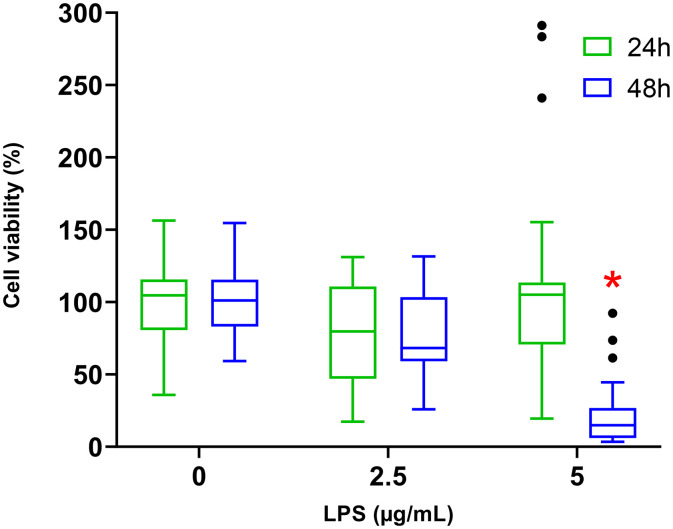
Viability of H9c2 cells by MTT assay, after 24 hours of exposure to 0 (n = 8); 2.5 (n = 4); 5 (n = 8) µg/mL of LPS or 48 hours with 0 (n = 5); 2.5 (n = 3); 5 (n = 5) µg/mL of LPS. Values are represented as medians, 1st quartile, and 3rd quartile. The Kruskal-Wallis test followed by Dunn’s post hoc test was used to compare the effects of each LPS concentration with the respective control at 24 or 48 hours. * indicates a significant difference compared to the control condition (*p-value < 0.05*).

Exposure of cells for 24 hours to 2.5 or 5 µg/mL of LPS did not lead to significant changes in cell viability compared to control cells (No LPS). However, after 48 hours of exposure to 5 µg/mL of LPS, a significant decrease of 77.7 ± 3.8% in cell survival (*p < 0.0001*) was observed compared to the control group (Fig 1).

### 3.2 Gene expression

**Fig 2:**
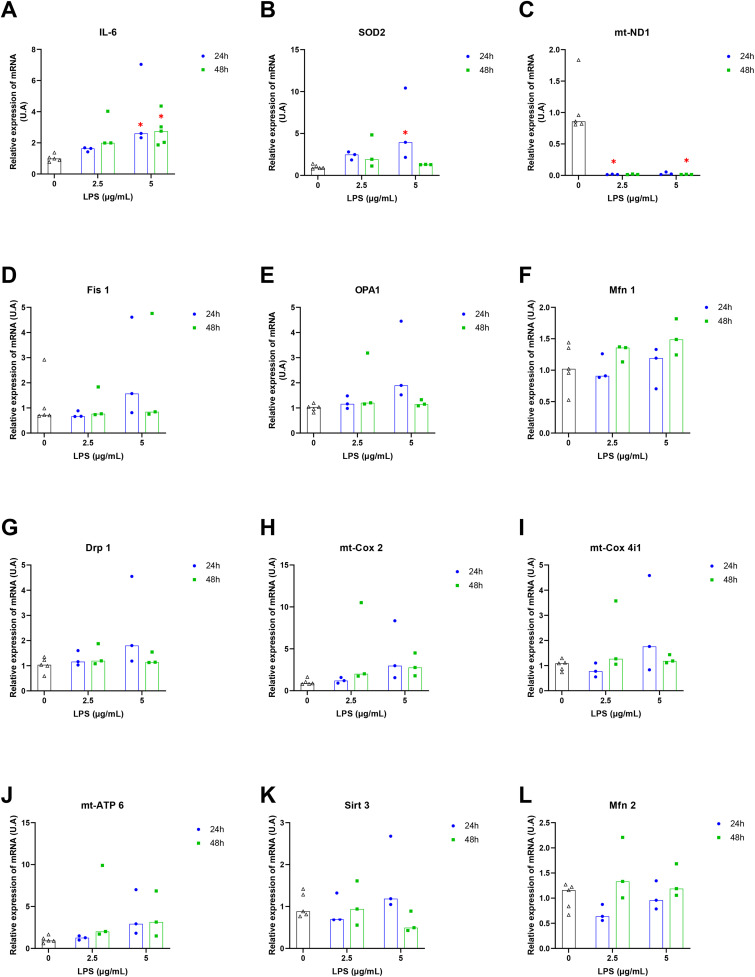
Relative expression of mRNA in H9c2 cells exposed to 2.5 and 5 µg/mL of LPS for 24 and 48 hours. Expression was normalized to β-actin mRNA. The 24 h and 48 h control groups were pooled due to the absence of significant differences between them. The analysis was performed using the ΔΔCT method for the groups: no LPS (n = 5); LPS 2.5 µg/mL (n = 3); LPS 5 µg/mL (n = 3 or 5). Results are presented as medians and individual values. The non parametric Kruskal-Wallis test followed by Dunn’s post hoc test was used to compare the effects of each LPS concentration with the control condition.* indicates p-value < 0.05 compared to the control condition.

An exposure to 5 µg/mL of LPS for 24 or 48 hours led to a significant overexpression of IL-6 transcripts compared to the control (Fig 2A). A 24-hour exposure to 5 µg/mL of LPS resulted in an increase of 4 ± 1.8 times (*p = 0.01*) compared to the control. Moreover, a 24-hour exposure to 5 µg/mL of LPS led to a significant overexpression (5.5 ± 2.5 times; *p = 0.03*) of *SOD2* transcripts compared to control cells (Fig 2B). Conversely, a significant underexpression of *mt-ND1* transcripts was observed compared to the control group (Fig 2C). For all experimental conditions, no significant changes in the expression of *Fis1* (Fig 2D), *OPA1* (Fig 2E), *Mfn1* (Fig 2F), *Drp1* (Fig 2G), *mt-Cox2* (Fig 2H), *mt-Cox4i1* (Fig 2I), and *mt-ATP6* (Fig 2J) transcripts were observed compared to the control condition. Overall, a high variability of data is observed after 24 hours of exposure to 5 µg/mL of LPS and 48 hours at 2.5 µg/mL of LPS.

### 3.3 Mitochondrial respiration and energetic metabolism

#### 3.3.1 Oxidative metabolism.

**Fig 3:**
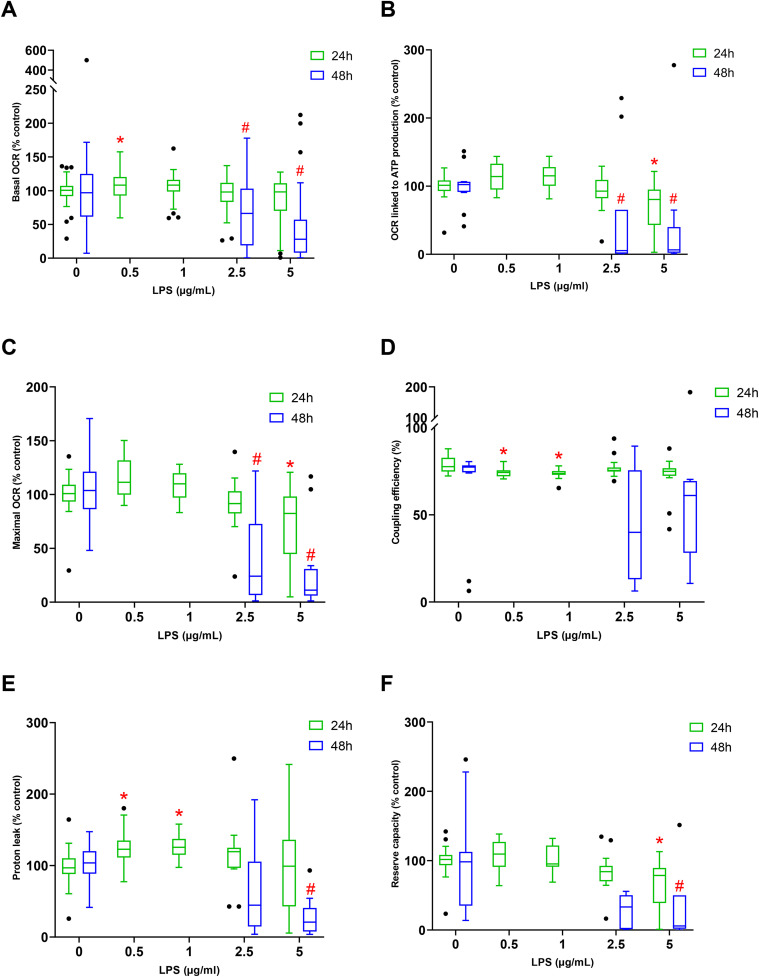
Mitochondrial respiration and energetic metabolism measured and calculated on H9c2 cells exposed without LPS for 24 hours (n = 6) or at 0.5 (n = 3); 1 (n = 3); 2.5 (n = 3); 5 (n = 4) µg/mL of LPS and for 48 hours at 0 (n = 3); 2.5 (n = 3); 5 (n = 3) µg/mL. Basal OCR (A), OCR related to ATP production (B), Maximal OCR (C), Coupling efficiency (D), Proton leak (E), Reserve capacity (3F). Values are represented as medians, 1st quartile, and 3rd quartile. The Kruskal-Wallis test followed by Dunn’s post hoc test was used to compare the effects of each LPS concentration with the respective control at 24 or 48 hours. * indicates p-value < 0.05 compared to the 24-hour control condition; # indicates p-value < 0.05 compared to the 48-hour control condition).

After 24 hours of exposure to LPS 0.5, a significant increase in basal OCR of H9c2 was observed compared to control cells. For example, an increase of 7.9 ± 2.4% in basal OCR was measured after 24 hours of exposure to 0.5 µg/mL of LPS (*p = 0.021*). This effect was no longer observed at higher concentrations of LPS (2.5 and 5 µg/mL). In contrast, after 48 hours of exposure to LPS, a decrease in basal OCR of 12.4 ± 4,0% and 55.8 ± 6.4% was observed at 2.5 and 5 µg/mL of LPS respectively (Fig 3A).

Exposure to 5 µg/mL of LPS for 24 hours, as well as exposure to 2.5 and 5 µg/mL LPS for 48 hours, significantly decreased OCR-linked ATP production and maximal OCR. For example, a decrease of 31.1 ± 9.0% (*p = 0.013)* and 28.5 ± 9.0% (*p = 0.02)* was respectively measured in OCR-linked ATP production and maximal OCR after 24 hours exposure to 5 µg/mL of LPS (Fig 3B and 3C). Whereas, a trend toward increased OCR-linked ATP production and maximal OCR was observed at 0.5 and 1 µg/mL of LPS.

Furthermore, a significant decrease in coupling efficiency was observed compared to the control group after 24 hours of exposure to 0.5 and 1 µg/mL of LPS (Fig 3D). For example, after 24 hours of exposure at 1 µg/mL of LPS, a decrease of 5.2 ± 0.7% is noted (*p = 0.002*).

After 24 hours of exposure, at 0.5 and 1 µg/mL of LPS a significant increase in proton leak was described compared to control cells. Indeed, exposure to 1 µg/mL of LPS resulted in an increase of 26.4 ± 4.3% (*p = 0.002*) in proton leak. Whereas after 48 hours of exposure to 5 µg/mL of LPS, a significant decrease was observed compared to control (Fig 3E).

After 24 and 48 hours of exposure to and 5 µg/mL of LPS, reserve capacity was significantly reduced compared to the control group (Fig 3F). After 24 hours of exposure, regardless of the LPS concentration, non-mitochondrial OCR was not significantly altered (data not shown).

#### 3.3.2 Glycolytic metabolism.

**Fig 4:**
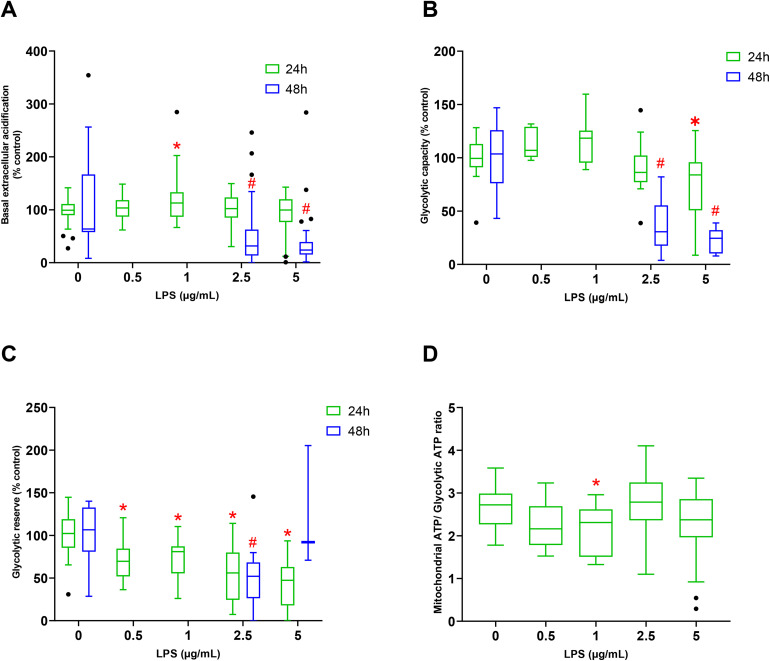
Parameters of glycolytic metabolism measured and calculated on H9c2 cells exposed without LPS for 24 hours (n = 6) or at 0.5 (n = 3); 1 (n = 3); 2.5 (n = 3); 5 (n = 4) µg/mL and for 48 hours with no LPS (n = 3) and at 2.5 (n = 3); 5 (n = 3) µg/mL. Basal extracellular acidification (4A), Glycolytic capacity (4B), Glycolytic reserve (4C); mitochondrial ATP/Glycolytic ATP ratio (4D). Values are represented as medians, 1^st^ quartile, and 3^rd^ quartile. The Kruskal-Wallis test followed by Dunn’s post hoc test was used to compare the effects of each LPS concentration with the respective control at 24 or 48 hours.* indicates *p*-value < 0.05 compared to the 24-hour control condition; ^#^ indicates *p-*value < 0.05 compared to the 48-hour control condition.

After 24 hours of exposure to 1 µg/mL of LPS, a significant increase (16.0 ± 4.8%; *p = 0.0038*) in extracellular acidification compared to the control group was observed. This effect was not observed at 2.5 and 5 µg/mL of LPS. However, after 48 hours of treatment with 2.5 and 5 µg/mL of LPS, extracellular acidification was significantly decreased. For example, by 51.0 ± 7.8% (*p < 0.0001*) after 48 hours exposure to 2.5 µg/mL of LPS compared to control (Fig 4A).

After 48 hours of treatment with 2.5 µg/mL of LPS, a significant decrease in glycolytic capacity was measured as well as after exposure to 5 µg/mL of LPS for 24 hours and 48 hours compared to the control group. Indeed, 24 hours of exposure to 5 µg/mL resulted in a significant decrease of 27.9 ± 8.2% (*p = 0.019*) (Fig 4B).

After 24 hours of exposure to LPS, all experimental conditions led to a significant decrease in glycolytic reserve compared to control (Fig 4C). A decrease of 24.4 ± 6.0% (*p = 0.041*) is observed at 1 µg/mL of LPS. This decrease was of 55.5 ± 8.2% *(p < 0.0001)* was observed at 5 µg/mL of LPS. After 48 hours of exposure to LPS at 2.5 µg/mL, glycolytic capacity was significantly decreased by 47.1 ± 13.8% (*p = 0.021*) compared to control. The results for glycolytic reserve after 48 hours of exposure to 5 µg/mL of LPS were not usable.

At 1 µg/mL of 24 h LPS exposure, the ratio between the mitochondrial ATP and the glycolytic ATP was significantly decreased by 20.6% (*p = 0.026*), respectively, compared to the control group. This effect was no longer observed at 2.5 and 5 µg/mL of LPS (Fig 4D).

#### 3.3.3 Substrate utilization.

**Fig 5:**
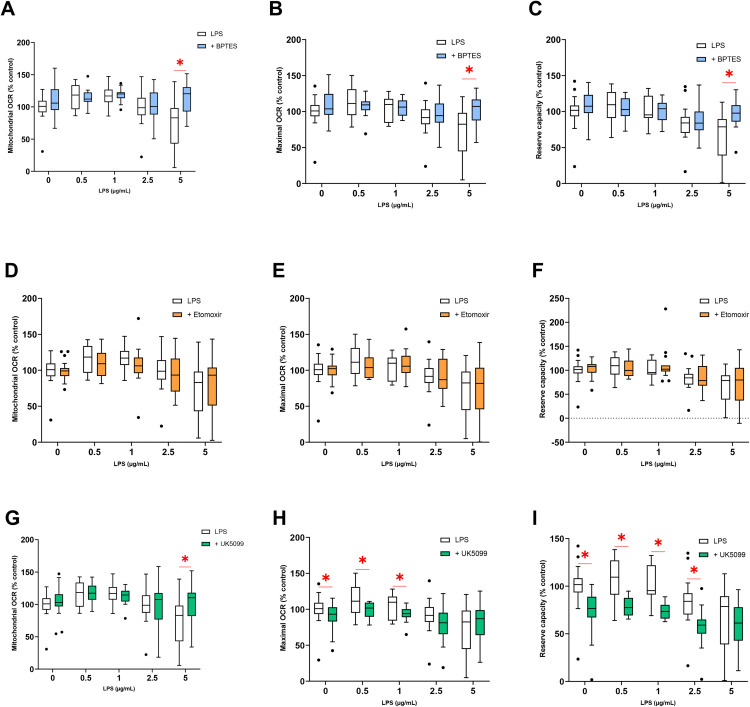
Parameters of OCR measured on H9c2 cells exposed to LPS for 24 hours in the presence of an amino acid pathway inhibitor: Mitochondrial OCR (A), Maximal OCR (B), Reserve capacity (C) or in the presence of an fatty acid pathway inhibitor (Etomoxir): Mitochondrial OCR (D), Maximal OCR (E), Reserve capacity (F) or in the presence of a glycolysis pathway inhibitor (UK 5099). Values are represented as medians, 1^st^ quartile, and 3^rd^ quartile. A Mann-Whitney test was used to compare each LPS concentration with inhibitor to the corresponding condition without inhibitor. * indicates *p-value < 0.05* compared to the same condition without the inhibitor.

The inhibition of the amino acid pathway by BPTES had no significant effect on the variations of mitochondrial OCR, maximal OCR, and reserve capacity induced by exposure to 0.5, 1, and 2.5 µg/mL of LPS. At 5 µg/mL of LPS, the inhibition of the amino acid pathway compensates for the decrease in mitochondrial OCR, maximal OCR, and reserve capacity caused by LPS (Fig 5A, 5B and 5C).

In the absence of LPS, the inhibition of the fatty acid pathway by etomoxir did not alter mitochondrial OCR, maximal OCR, or reserve capacity. Regardless of the experimental condition, the inhibition of the fatty acid pathway by etomoxir did not compensate for the changes in mitochondrial OCR, maximal OCR, and reserve capacity induced by LPS, as described in part 3.3.1 (Fig 5D, 5E and 5F).

For all experimental conditions except 5 µg/mL of LPS, the inhibition of the glycolysis pathway by UK5099 had no significant effect on the previously described variations in mitochondrial OCR (part 3.3.1). At 5 µg/mL of LPS, the inhibition of glycolysis compensates for the decrease in mitochondrial OCR caused by LPS (*p = 0.041*) (Fig 5G). In the absence of LPS and at 0.5 and 1 µg/mL of LPS, the inhibition of glycolysis led to a significant decrease in maximal OCR. For example, in the absence of LPS, the inhibition of glycolysis resulted in a significant decrease of 8.7 ± 3.0% (*p = 0.014*). This effect of inhibition was not observed at 2.5 and 5 µg/mL of LPS (Fig 5H). For all experimental conditions, except 5 µg/mL of LPS, the inhibition of glycolysis induced a significant decrease in reserve capacity. In the absence of LPS, a decrease of 26.0 ± 3.8% (*p < 0.0001*) in reserve capacity was noted in the presence of UK5099 while the inhibition of glycolysis at 1 µg/mL of LPS decreased the reserve capacity by 28.1 ± 2.1% (*p = 0.0001*)) (Fig 5I).

## 4 Discussion

In the context of sepsis, when an uncontrolled systemic inflammatory response to infection can alter the activities of tissues and organs, LPS was used to induce a direct activation of inflammatory intracellular pathways observed in SIMD to investigate the induced mitochondrial dysfunction. Our results showed that while 2.5 and 5 µg/mL of LPS for 24 hours did not induced cytotoxicity, the exposure to 5 µg/mL of LPS for 48 hours led to 77.7% decrease in cell viability. Gene expression revealed an overexpression of *IL-6* and *SOD2* transcripts with consistent underexpression of *mt-ND1*. Our inflammatory model was validated by those results. The study of mitochondrial respiration revealed an increase at lower LPS concentrations but a decrease at higher LPS concentrations. Glycolytic metabolism also increased at lower LPS levels while decreasing at higher concentrations. Our results revealed two distinct metabolic profiles in response to LPS.

The reduction of survival rate in H9c2 cells after 48 h of exposure to LPS at 5 µg/mL is in accordance with the literature, despite the wide range of responses to LPS varying from 0.1 to 10 µg/mL

In our experiment, overexpression of Il-6 transcripts, was observed. This overexpression supports validation of our experimental model. In our model, as in other studies [24,25], LPS triggered inflammatory signalling pathways and cytokine secretion. Our results suggest an inflammatory response in H9c2 cells, likely leading to oxidative stress due to an imbalance between radical defence systems and ROS production. The increase in *SOD2* mRNA expression after 24 hours of LPS exposure at 2.5 µg/mL supports this hypothesis and the establishment of oxidative stress, likely due to inflammation and secreted cytokines like IL-6. The same results (overexpression of *IL-6* and *SOD2*) were observed in a neuroinflammation model (BV2 cells) exposed to 200 ng/mL of LPS for 5.5 hours [26]. *SOD2* mRNA overexpression was also demonstrated in H9c2 and AC16 cardiomyocytes exposed to 10 µg/mL of LPS for 24 hours [27]. When redox balance is not restored, inflammation leads to cellular damage, including mitochondrial dysfunction. To maintain functional integrity and energy needs, damaged mitochondria are removed through quality control mechanisms, including mitochondrial fission and fusion. Thus, we have studied the gene expression of fission genes (*Fis1* and *Drp1*) and fusion genes (*OPA1, Mfn1,* and *Mfn2*). Even though, we did not observed significant change in expression of *OPA1, Mfn1, Drp1* or *Fis1* after LPS exposure, a trend of *Mfn2* underexpression after 24 hours of exposure to 2.5 µg/mL of LPS was observed., Literature indicates that LPS can increase mitochondrial fission mechanisms to contain mitochondrial damage and decrease fusion to maintain equilibrium between these processes. For example, in a murine cardiac cell model (HL-1), LPS (10 mg/mL) significantly increased fission gene expression (*Drp1* and *Fis1*) and decreased fusion gene expression (*Mfn2* and *OPA1*) [28]. Further experiments are required to confirm this trend. In our study, the gene expression of subunits of various mitochondrial transport chain complexes was also investigated. An underexpression of *mt-ND1* transcripts at 24 and 48 hours of LPS exposure was observed whereas no change in expression of *mt-Cox2, mt-Cox4i1* and *mt-ATP6* was shown. As in our study, in an *in vitro* model of acute kidney injury induced by 4 µg/mL of LPS (HK-2 cells), a decrease in *mt-ND1* mRNA expression was demonstrated The downregulation of *mt-ND1*, the gene encoding complex I suggest metabolic alterations and mitochondrial dysfunction in our study. In summary, based on our results, LPS is primarily recognized by the TLR4 receptor activating both signalling pathways leading to the activation of the NFkB transcription factor. This factor promotes the secretion of pro-inflammatory cytokines, such as IL-6, disrupting the redox balance and triggering oxidative stress. This stress is partially managed by SOD2. At high concentrations of LPS (5 µg/mL) and prolonged exposure times of 48 hours, inflammation and oxidative stress exceed the adaptive capacities of the cell, resulting in deleterious cellular damage, particularly mitochondrial, which disrupts cellular and mitochondrial function.

In our study, an increase in basal OCR for cells exposed to low concentration of LPS was observed. This suggests that LPS leads to an increase in the basal metabolic needs necessary to maintain essential cellular functions. The trend toward increased maximal OCR at low LPS concentrations may indicate a better efficiency of the electron transport chain and a greater oxidative metabolic efficiency. Moreover, the trend toward enhanced reserve capacity at low LPS concentrations also suggests effective mitochondria that are potentially capable of adapting to metabolic disturbances. Similar results have been observed in a model of synovial fibroblast cells from rheumatoid arthritis patients exposed to 1 µg/mL of LPS for 24 hours [29] and in a model of primary neutrophils extracted from healthy volunteers and exposed to 0.1 µg/mL of LPS for 4 or 8 hours [30]. These observations may reflect a phenomenon of mitohormesis, whereby moderate exposure to mitochondrial stress induces the establishment of protective adaptive responses beneficial to the cell. Nevertheless, in our study, these adaptation to LPS seem to be exceeded at high concentrations of LPS, potentially causing cellular and mitochondrial damage. Indeed, from 2.5 µg/mL of LPS and when the cells are faced with a simulated high energy demand (FCCP), our results showed a decrease in maximal OCR and reserve capacity. The same results were observed in a model of porcine intestinal epithelial cells (IPEC-J2) exposed for 48 hours to 40 ng/mL of LPS [31] or in microglial cells (BV2) by [32]. These results may be partly explained by a decrease in the efficiency of the electron transport chain (ETC), particularly in the enzymatic activity of the different ETC complexes in response to LPS [33].

Our study reveals that 24 hours of LPS exposure leads to increased proton leak and decreased coupling efficiency at low concentrations. Similarly, an increase in proton leak have been observed in a rat model of inflammatory pathology [34]. It may be indicative of mitochondrial damage as it is associated with a decrease in ATP concentration and mitochondrial membrane potential. However, by decreasing mitochondrial membrane potential, it can also be beneficial and regulate ROS production. Indeed, proton leak can be induced by UCPs 1–3, which are directly activated by superoxide dismutase (SOD) to induce mitochondrial membrane depolarization and reduce ROS production [35]. However, in our model, further investigations are needed to determine if the increase is beneficial or deleterious. The uncoupling observed may be linked to the partial opening of the mitochondrial permeability transition pore, resulting in mitochondrial membrane potential loss [36,37]. Ultimately, this uncoupling can lead to reduced ATP levels and cell death [38].

Our results suggest that LPS induces metabolic reprogramming. Indeed, we observed a decrease at low LPS concentrations of the ratio determining the contribution of oxidative phosphorylation and glycolysis to ATP and pointing towards a glycolytic phenotype in H9c2 cells [39]. [39] found similar results in macrophages exposed to 100 ng/mL of LPS for 12 hours, showing significant increases in glycolysis and a reduced oxidative/glycolytic metabolism ratio, confirming metabolic reprogramming towards a glycolytic phenotype. Several studies demonstrated that this metabolic reprogramming involves multiple pathways [40,41]. Moreover, in our study, a decrease in glycolytic metabolism was observed at high concentrations of LPS (2.5 and 5 µg/mL), similar to oxidative metabolism. This shows that high concentrations of LPS and/or prolonged exposure likely reduced metabolic flexibility due to LPS exposure and cause deleterious cellular damage or glycolytic disturbances involving mediators. Similar findings were reported in primary cardiomyocytes from TBMIM6 mice injected with LPS 24 hours prior to experimentation [42].

Our results mainly show that H9c2 use glucose as the primary substrate in cases of high metabolic demand. Indeed, when glycolysis is inhibited, the control group cells and those exposed to low concentrations of LPS can no longer meet the high metabolic needs. Thus, glycolysis, which provides reduced equivalents for oxidative phosphorylation, seems essential during significant metabolic needs, and its inhibition does not appear to be compensated by amino acid or fatty acid pathways. Van Wyngene *et al* explain that glycolysis allows for rapid ATP generation, thus providing immediate energy through the action of glycolytic enzymes, which can be crucial in response to stress, unlike oxidative phosphorylation, which is a slower and more complex process [43]. The absence of compensation by the amino acid pathway is consistent, as they contribute only in very small proportion to ATP synthesis. In a context of normal energy demand (without LPS), the inhibition of the glutamine, fatty acid, or glycolytic pathways does not lead to changes in mitochondrial O₂ consumption, suggesting compensation by the remaining pathways. In contrast, this compensation is limited to mitochondrial O₂ consumption when glycolysis is inhibited and is not observed at all when the fatty acid pathway is blocked.

In conclusion, in our study, we have described the direct metabolic (mitochondrial and glycolytic) effects of LPS on activity in H9c2 cells that vary based on exposure time and concentration. A phenomenon of mitohormesis occurs in response to LPS exposure, where H9c2 cells develop adaptive mechanisms to combat the stress caused by LPS. This is reflected in increased oxidative and glycolytic metabolism, as well as a metabolic reprogramming towards a glycolytic phenotype. However, when these adaptive mechanisms are overwhelmed, mitochondrial and cellular damage ensues, leading to a decrease in metabolism. In the context of SIMD, it would be interesting to go further in the analysis of cardiac metabolic disturbances coupling LPS direct effects with proinflammatory cytokines simulating a systemic inflammation in order to understand the combined direct effects of LPS with a simulated systemic inflammation.

There are also some limitations to this study. One notable limitation is the use of immature cardiomyocytes. Although H9c2 cardiomyoblasts are among the most commonly used cell lines in the literature for studying various cardiac syndromes, they, like any *in vitro* model, have inherent limitations. As immortalized cells, they have lost some physiological characteristics, such as contractile function. Alternative models, such as primary cardiomyocytes or induced pluripotent stem cell (iPSC)-derived cardiomyocytes, can offer more physiologically relevant data. However, these models are more complex to maintain in culture and are less cost-effective. In addition, the use of primary cardiomyocytes requires animal sacrifice, which poses ethical concerns.

It is also important to note that none of these *in vitro* models, including H9c2 cells, can fully reproduce the complexity of a whole organism. Nevertheless, to support the validity of our findings, our results were confirmed using a primary cardiomyocyte model extracted from mice by [42].

## Supporting information

S1 Data The supplementary file contains the raw data from the different assays: viability assay (optical density, Sheet 1), gene expression analysis (threshold cycle values, Sheet 2), oxygen consumption measurements over time (OCR, Sheets 3–6), and extracellular acidification rate measurements over time (ECAR, Sheets 7–9). (XLSX)
